# Proregenerative Microenvironment Triggered by Donor Mesenchymal Stem Cells Preserves Renal Function and Structure in Mice with Severe Diabetes Mellitus

**DOI:** 10.1155/2015/164703

**Published:** 2015-06-08

**Authors:** Fernando Ezquer, Maximiliano Giraud-Billoud, Daniel Carpio, Fabián Cabezas, Paulette Conget, Marcelo Ezquer

**Affiliations:** ^1^Centro de Medicina Regenerativa, Facultad de Medicina Clínica Alemana, Universidad del Desarrollo, Avenida Las Condes 12438, Santiago, Chile; ^2^Laboratorio de Fisiología (IHEM-CONICET) and Instituto de Fisiología, Facultad de Ciencias Médicas, Universidad Nacional de Cuyo, Casilla de Correo 33, Mendoza, Argentina; ^3^Instituto de Anatomía, Histología y Patología, Facultad de Medicina Universidad Austral de Chile, Isla Teja s/n, Valdivia, Chile; ^4^Facultad de Ciencias Biológicas, Universidad Andrés Bello, Avenida Los Leones 745, Santiago, Chile

## Abstract

The aim of our work was to evaluate, in an animal model of severe diabetes mellitus, the effect of mesenchymal stem cells (MSCs) administration on diabetic nephropathy (DN) progression. After diabetes induction, one group of mice received the vehicle (DM) and other group received a single dose of MSCs (DM + MSCs). DM + MSCs mice showed a significant improvement in functional parameters of the kidney compared with untreated mice. While DM mice presented marked histopathological changes characteristics of advanced stages of DN (fibrosis, glomerulosclerosis, glomerular basement membrane thickening, capillary occlusion, decreased podocyte density, and effacement of foot processes), DM + MSCs mice showed only slight tubular dilatation. The renoprotection was not associated with an improvement in diabetic condition and very low number of donor cells was found in the kidney of DM + MSCs mice, suggesting that renoprotection could be mediated by paracrine effects. Indeed, DM + MSC mice presented increased renal proliferation index, decreased renal apoptotic index and the restoration of proregenerative factors, and anti-inflammatory cytokines levels. Moreover, macrophage infiltration and oxidative stress damage were also reduced in DM + MSCs mice. Our data demonstrate that MSC administration triggers a proregenerative microenvironment in DN kidney, which allows the preservation of the renal function even if diabetes was uncorrected.

## 1. Introduction

Diabetic nephropathy (DN) is the major microvascular complication in diabetic patients and it remains the leading cause of chronic kidney disease, responsible for approximately 50% of all cases of end-stage renal disease worldwide [1]. The development of DN is stimulated by sustained hyperglycaemia [2], and its key pathologic features include gradual thickening of the glomerular basement membrane (GBM) and glomerular hypertrophy accompanied by mesangial matrix expansion with accumulation of several matrix proteins, such as collagen type I, laminin *β*1, and fibronectin [2]. These molecules contribute to the reduction in the glomerular filtration surface area and to an increase in tubular interstitial fibrosis and glomerulosclerosis. Clinically, there is a decline in glomerular filtration rate with progressive increase in urinary albumin excretion that ultimately leads to end-stage renal failure [3].

Hyperglycaemia induces cellular changes in various cell types present in the kidney. In the normal glomerulus, the podocytes (glomerular epithelial cells) have an arborized phenotype, and its terminal branches or foot processes cover the outer wall of the glomerular capillaries. During proteinuric stages, the podocyte typical morphology is lost and is converted into a flatter epithelial cell, a process named as “foot process effacement.” In this process, the cytoskeleton that normally supports the delicate architecture of the foot processes is condensed at the basal side of the flattened podocytes, altering its functionality [4]. Therefore, podocyte injury is associated with the development of albuminuria and the appearance of more severe glomerular structural abnormalities [4].

Throughout diabetes onset, high levels of blood glucose induce both oxidative stress and inflammation, which are intimately linked with the development of DN [5–7]. Oxidative stress is caused by an imbalance between the production of reactive oxygen species (ROS) and the biological system's ability to readily detoxify the reactive intermediates or repair the resulting damage. When this occurs, oxidation of essential macromolecules including proteins, lipids, carbohydrates, and DNA is produced, leading to the apoptosis of damaged cells [6].

Higher ROS levels can also induce the production of inflammatory cytokines, which stimulate the production of free radicals exacerbating DN progression [5]. Therefore, intensive glycemic control is the main intervention to reduce oxidative stress.

Transforming growth factor beta (TGF-*β*) is a multifunctional cytokine that plays a critical role in the pathogenesis of DN by stimulation of the synthesis of extracellular matrix molecules including collagen type I, fibronectin and laminin *β*1, leading to renal fibrosis [8].

Stem cell therapy has been envisioned as a novel strategy for various diseases since it has the potential to be more effective than single-agent drug therapies [9]. Multipotent mesenchymal stromal cells, also referred as mesenchymal stem cells (MSCs), are a population of self-renewable and undifferentiated cells present in the bone marrow and other mesenchymal tissues of adult individuals [10]. MSCs are attractive candidates for renal repair, since nephrons are of mesenchymal origin and because stromal cells are of crucial importance for signalling, leading to differentiation of both nephrons and collecting ducts [11].

Several studies have shown that the administration of MSCs leads to the amelioration of acute and chronic kidney injury [12]. In those reports, immune modulation and antiapoptotic effects have been associated with the therapeutic effect, suggesting that MSCs can act through paracrine mechanisms [13, 14]. Additionally, MSC-based therapies have been considered to be promising strategies to treat patients with diabetes mellitus and its main complications [15]. We and others have demonstrated that the systemic administration of MSCs into immunologically induced type 1 diabetic mice reduced microalbuminuria and preserved normal renal histology [16–20]. However, animal models used only developed the earlier harbingers of DN; furthermore in these studies, it is difficult to distinguish whether restoration of kidney function is based on direct effect of transplanted cells on renal tissue or indirectly, through improvement of pancreatic endocrine function. To answer this question and taking into account that hyperglycaemia is the driving force for the development of DN, previously, we used a severe diabetic animal model induced by the administration of a single high dose (200 mg/kg) of streptozotocin (STZ) in C57BL/6 mice [21]. This protocol generates an irreversible and massive destruction of insulin-producing cells and severe hyperglycaemia, leading to a rapid progression of renal failure and the appearance of most of the characteristics DN pathognomonic signs [21–23]. In this model, multiple systemic administrations of MSCs did not reverse hyperglycaemia but protect kidneys from progression to macroalbuminuria and prevent the development of marked histological alterations [21].

The aim of the present work is to further characterize the therapeutic effect of MSC administration in an animal model of severe diabetes mellitus, particularly regarding renal failure.

For this purpose, eight weeks after the massive cytotoxic destruction of insulin-producing cells, severe diabetic mice were separated into two groups: one received the vehicle (DM mice) and the other 0.5 × 10^6^ MSCs (DM + MSCs mice). Two and eight weeks later, the function, histopathology, ultrastructure, cell proliferation, protein profile, gene expression, oxidative stress, and inflammation of kidneys were examined. Donor MSCs biodistribution was also assessed.

## 2. Material and Methods

### 2.1. Animals

C57BL/6 and C57BL/6-Tg (ACTB-EGFP) 1Osb/J mice (Jackson Laboratory) were housed at constant temperature and humidity, with a 12 : 12 h light-dark cycle and unrestricted access to a standard diet and water. When required, animals were slightly anaesthetized with sevoflurane (Abbott) or ketamine (Drag Pharma) plus xylazine (Centrovet). All procedures were in accordance with institutional and international standards for the human care and use of laboratory animals [Ethic Committee of Facultad de Medicina Clinica Alemana, Universidad del Desarrollo and Animal Welfare Assurance Publications A54427-01, Office for Protection from Research Risks, Division of Animal Welfare, NIH (National Institute of Health), Bethesda, MD, USA].

### 2.2. Severe Diabetes Induction

Eight-week-old male C57BL/6 mice were slightly anesthetized and received intraperitoneally 200 mg/kg STZ (Calbiochem) immediately after dissolving in 0.1 M citrate buffer at pH 4.5 (DM mice) or citrate buffer only (Normal mice). This protocol of STZ administration causes massive cytotoxic destruction of insulin-producing cells, generating a severe hyperglycemic condition, which accelerates the appearance of the complications associated to diabetes, particularly DN [21–23].

### 2.3. MSC Isolation and* Ex Vivo* Expansion

Eight- to 10-week-old male C57BL/6 or C57BL/6-Tg (ACTB-EGFP) 1Osb/J mice were sacrificed by cervical dislocation. Bone marrow cells were obtained by flushing femurs and tibias with sterile phosphate-buffered saline (PBS). After centrifugation, cells were resuspended in *α*-minimum essential medium (*α*-MEM) (Gibco) supplemented with 10% selected fetal bovine serum (Gibco) and 0.16 mg/mL gentamicin (Sanderson Laboratory) and plated at a density of 1 × 10^6^ nucleated cells/cm^2^. Nonadherent cells were removed after 72 h by media change. When foci reached confluence, adherent cells were detached with 0.25% trypsin, 2.65 mM EDTA, centrifuged, and subcultured at 7000 cells/cm^2^. After 2 subcultures, adherent cells were characterized according to their adipogenic and osteogenic differentiation potential and immunophenotypified as previously described [24, 25].

### 2.4. MSC Administration

Eight weeks after diabetes induction 0.5 × 10^6^ MSCs or 0.5 × 10^6^ MSCs^GFP^ were resuspended in 0.2 mL of 5% mice plasma (vehicle) and administered via the tail vein to slightly anaesthetized mice (DM + MSCs mice). Untreated animals received 0.2 mL of vehicle (DM mice).

### 2.5. Sample Collection

After four hours of fasting, mice were sacrificed and blood samples were collected. Kidneys were quickly removed, decapsulated, weighted, and dissected into two parts, one of which was immediately frozen in liquid nitrogen and stored at −80°C for molecular biological studies, while the other was stored in 4% paraformaldehyde for histopathological analysis.

For 24 h urine collection, animals were placed individually in metabolic cages for 24 hours, and urine was collected and gravimetrically measured.

### 2.6. Physical and Biochemical Assessment

Body weight, kidney weight, and biochemical parameters were measured eight weeks after diabetes induction (before MSC administration) and two weeks and eight weeks after MSC administration. Blood glucose levels were measured with the glucometer system Accu-Check Performa (Roche Diagnostic). Plasma insulin levels were assayed using a mouse-specific insulin ELISA kit (Ultrasensitive Mouse Insulin ELISA; Mercodia). Serum triglycerides and cholesterol levels were determined in the Architect c8000 autoanalyzer (Abbot). The renal mass index, determined by the ratio of kidney weight to body weight, was also calculated.

### 2.7. Renal Function Measurements

Urine samples were centrifuged to remove any suspended particle and the supernatant was used to detect albumin concentration using a mouse-specific albumin ELISA kit (Albuwell, Exocell). Urine creatinine concentration was determined by the Creatinine Companion kit (Exocell). Excretion of urinary albumin was determined using albumin to creatinine ratio.

Blood samples were centrifuged at 2000 ×g for 10 min, plasma creatinine levels were determined using a mouse-specific enzymatic kit (Mouse Creatinine Assay, Crystal Chem) and blood urea nitrogen (BUN) using a quantitative colorimetric kit (QuantiChrom Urea Assay Kit, BioaAssay Systems).

### 2.8. Renal Histological and Immunofluorescence Analysis

Periodic acid Schiff (PAS) staining was performed on 4-*μ*m sections of paraffin-embedded slices. Stained slices were used to capture 50 photomicrographs (Leica DM2000 microscope and Leica DFC295 digital camera) of glomeruli containing vascular pole and/or urinary pole to determine glomerular corpuscle area, glomerular tuff area, and mesangial expansion in each image using Image-Pro Plus 4.5.1 software (Media Cybernetics). Masson's trichrome staining was used to evaluate the total area of collagen fibbers in the glomerular and in the tubulointerstitial area (20 photomicrographs each) and the leukocyte infiltrate. The fields were randomly chosen and analyzed in blind by the same specialist nephrologist. Leukocyte infiltration was graded, from 0 to 3; (0 = absent or infrequent, 1 = sporadic, scattered and ungrouped, 2 = focalized in dense groups, and 3 = dense foci + scattered presence) [26].

A standard point-counting method was employed to quantitate the collagen deposition [14, 27, 28]. Under high magnification of the 40x objective, 20 consecutive nonoverlapping fields, in each kidney, were observed. Trichrome-positive points falling on glomeruli, Bowman's capsules or vessels, were excluded, while interstitial trichrome-positive points were counted for evaluation. The index of interstitial collagen volume was defined as the number of trichrome-positive points in every 2000 points evaluated.

Tubular damage was graded, from 0 to 4, according to the presence or not of dilatation, protein cylinders, atrophy, interstitial fibrosis, and leukocyte infiltration (0 = no changes, 1 = changes affecting <25% of the sample, 2 = changes affecting 25% to 50% of the sample, 3 = changes affecting 50% to 75% of the sample, and 4 = changes affecting 75% to 100% of the sample) [29].

Renal markers immunoreactivity was evaluated by confocal microscopy. Sections of 4 *μ*m thick were deparaffinized in Neo-Clear (Merck), rehydrated, and washed in Tris-buffered saline (TBS). Antigen retrieval was performed by incubation in 10 mM citrate buffer pH 6, in a boiling water bath for 30 min. Sections were blocked with 2% bovine serum albumin (Sigma) in TBS and incubated with the primary antibodies (WT-1 sc-192, podoplanin sc-134483 [Santa Cruz Biotechnology]; F4/80 ab74383, Ki-67 ab15589, *α*-SMA ab5694 [Abcam]; GFP-D5.1XP [Cell Signaling]) in Signal Stain Ab diluent (Cell Signaling) at 4°C overnight. After washing, sections were incubated with secondary antibody (anti-rabbit IgG Fab2 Alexa Fluor555, Molecular Probes) at room temperature for 1 h and nuclei were counterstained with 4′,6-diamino-2-fenilindol (DAPI) (BioChemica A1001, AppliChem). Sections were examined with the Fluoview FV10i confocal microscope (Olympus).

F4/80 positive cells in renal tissue were quantitatively assessed in 30 random sections per animal and expressed as F4/80 cells/cross sections [30].

In normal kidneys, *α*-SMA expression is confined to the smooth muscle cells of efferent and afferent arterioles and it is never observed in glomeruli [31, 32]. Glomerulosclerosis was defined as the presence of prominent *α*-SMA immunoreactivity in the mesangial area. Glomerulosclerosis index was quantified by determining the percentage of glomeruli exhibiting intense *α*-SMA staining in the glomerular tuft [33]. One hundred consecutive glomeruli were examined for each animal and analyzed in blind by the same specialist nephrologist.

Glomerular podoplanin fluorescence area was analyzed measuring the relation between podoplanin staining and glomerular tuft area [34]. Slides stained for WT-1 were used for morphometric analysis of the number of podocytes per glomerular volume. WT-1-positive nuclei per glomerular cross-section were counted and the glomerular volume per podocyte was calculated according to a previously validated method [34]. In all evaluations at least 50 glomeruli per animal were randomly selected and analyzed, and negative controls without the primary antibody were used to observe tissue background. Measurements of surface glomeruli area and the immunoreactivity analysis were made using the ImageJ 1.34 software (NIH, http://imagej.nih.gov/ij/).

### 2.9. Renal Ultrastructural Analysis

Small fragments of kidney tissue were fixed in 2% glutaraldehyde (Sigma), then post-fixed with 1% osmium tetroxide (Ted Pella Inc.), embedded in EMBed-812 resin (EMS, Hatfield), cut, stained, and analyzed under transmission electron microscopy (Philips Tecnai 12 Bio TWIN) at 80 kV, with standard magnifications between ×2,500 and ×16,500.

The electron micrographs of every animal were analyzed by setting the percentage of capillary loops with foot process effacement usually along with microvillous transformation of the podocyte and irregularities of the outer surface of the glomerular basement membrane. Then, a comparison was made between micrographs of the same magnification in the different groups of animals.

Images were analyzed in blind by a specialist pathologist.

### 2.10. Renal Gene Expression Analysis

Expression levels of type I collagen, TGF-beta1, laminin-beta1, fibronectin, nephrin, bFGF, EGF, HGF, IL-4, IL-10, and GAPDH from kidney samples were assessed by quantitative qRT-PCR. For this, total RNA was purified using TRIzol (Invitrogen) and quantified spectrophotometrically (260 nm). One *μ*g of total RNA was used for reverse transcription. Real time PCR was performed in a final volume of 20 *μ*L containing 50 ng of cDNA, PCR LightCycler-DNA Master SYBRGreen reaction mix (Roche), 3 mM MgCl_2_, and 0.5 *μ*M of each specific primer (see Supplementary Table 1 in Supplementary Material available online at http://dx.doi.org/10.1155/2015/164703), using a Light-Cycler thermocycler (Roche). To ensure that amplicons were from mRNA and do not from genomic DNA amplification, controls without reverse transcription were included. Amplicon validation was performed based on its size and melting point. Relative quantification was performed by the ΔΔCT method. The mRNA level of each target gene was normalized against the mRNA level of GAPDH and expressed as fold of change* versus* Normal mice.

### 2.11. Quantification of Renal Mitotic and Apoptotic Indexes

Mitotic index was determined by immunohistofluorescence using an anti-Ki-67 antibody. Positive nucleus indicates cell cycle activity (G1, S, G2, or M phase). Apoptotic cells in kidney tissue slices were visualized using the DeadEnd Fluorometric TUNEL System (Promega), following the manufacturer's protocol. The nuclei were counterstained with DAPI and fluorescence was evaluated by confocal microscopy.

For the quantification of Ki-67 and TUNEL positive cells, at least 30 glomeruli and 30 tubules sections were examined for each mouse, and the number of positive stained nuclei per total cell nuclei was determined. Data were expressed as level of change* versus* Normal mice.

### 2.12. Glomerular Fluorescein Angiography

Mice were injected by tail vein with 200 *μ*L of PBS containing 10 mg of 2 × 10^6^ molecular weight fluorescein-dextran (Sigma). Five minutes later, mice were euthanized and kidneys were fixed in 4% paraformaldehyde. Four *μ*m kidney slices were observed and photographed under confocal microscopy, and at least 50 glomeruli per animal were analyzed. The fluorescence area, representative of glomerular capillary lumen area, of each image was quantified and normalized to the glomerular tuft area [35], using the ImageJ 1.34 software, NIH.

### 2.13. Assessment of Renal Oxidative Stress and Antioxidant Capacity

#### 2.13.1. Total Reactive Oxygen Species

Kidney slides were incubated with 10 *μ*M 2,7-dichloro-dihydro-fluorescein diacetate (H_2_DCFDA) (Invitrogen) in PBS containing 5 mM Hepes for 60 min at 37°C. H_2_DCFDA is a nonfluorescent molecule that in contact with ROS (hydrogen peroxide, peroxynitrite, and hydroxyl radicals) is rapidly converted into a highly fluorescent derivate. Kidney slides were mechanically lysed in RIPA buffer and fluorescence was measured in a fluorometer (Turner) with excitation of 450 nm and emission of 530 nm. Data were presented as fluorescent units per mg of renal protein.

#### 2.13.2. Lipid Oxidative Level

Lipid peroxidation was determined in urine samples measuring isoprostane levels. Isoprostanes are a type of eicosanoids produced nonenzymatically through the oxygen radical peroxidation of tissue phospholipids and lipoproteins. Isoprostanes appear in normal urine samples but are elevated by oxidative stress in renal tissue [36]. Urine samples were acidified to pH 3.0 by adding 1/10 v/v of 1 N HCl and further diluted in sample diluent. Level of 8-iso-prostaglandin F2*α* was determined using the OxiSelect 8-isoPGF2*α* ELISA kit (Cell Biolabs, Inc.) according to the manufacturer's instructions.

#### 2.13.3. Protein Oxidative Damage

3-Nitrotyrosine modification of proteins is a well-established marker of protein damage by oxidative stress [37]. Kidney slides were homogenized with an extraction buffer supplemented with protease inhibitor cocktail (Thermo) and protein oxidative damage was measured with the 3-Nitrotyrosine ELISA kit (Abcam) according to the manufacturer's instructions.

#### 2.13.4. DNA Oxidative Damage

The formation of 8-hydroxy deoxyguanosine (8-OHdG) is a ubiquitous marker of oxidative stress and its urinary excretion is associated with renal DNA oxidative damage [38]. Urinary level of 8-OHdG was evaluated by the OxiSelect Oxidative DNA Damage ELISA kit (Cell Biolabs, Inc) in urine samples, according to the manufacturer's instructions.

#### 2.13.5. Total Antioxidant Capacity

Kidney slides were homogenized with an extraction buffer supplemented with protease inhibitor cocktail (Thermo). Total antioxidant capacity was measured in tissue homogenate using the Antioxidant Assay kit (Cayman Chemicals) [39] according to the manufacturer's instructions.

### 2.14. Quantification of Systemic Cytokines and Growth Factors

Cytokines (IL-1*β*, IL-4, IL-6, IL-10, IL-13, and TNF-*α*) and growth factors (EGF, ET1, b-FGF, HGF, and VEGF) were assessed in 25 *μ*L of plasma, using the MAGPMAG 24 k and MCYTOMAG 70 k assay kits (Luminex, Milliplex MAP, Millipore), respectively, according to the manufacturer's instructions. Plates were read on a Luminex 200 (Luminex Corp.) and analyzed with the Milliplex Analyst software (VigeneTech Inc.).

### 2.15. Statistical Analysis

Data are presented as mean ± S.E.M. To determine the statistical significance of intergroup differences, Krustal-Wallis test was used to compare mean values among all groups, and Mann-Whitney *U* test was used to compare mean values between two groups. *p* < 0.05 was considered as statistically significant.

## 3. Results

Severe diabetes mellitus was induced in C57BL/6 mice by the injection of a single dose of 200 mg/kg STZ. This protocol causes rapid and massive destruction of insulin-producing cell. A discrete acute nephrotoxic effect has been observed after diabetes induction, with any evidence of glomerular or tubular histopathological alterations [22, 23]. Diabetic mice were maintained without insulin supplementation to allow for the progression of severe diabetes mellitus and the appearance of its complications.

Eight weeks after diabetes induction mice were hyperglycemic, polyuric, and hypoinsulinemic (Table 1). Compared to normal mice they presented reduced body weight and increased serum cholesterol, serum triglycerides, and kidney weight (Table 1). In this condition, animals were randomly assigned into two groups: one group that received the vehicle (DM mice) and another group that received a single dose of 0.5 × 10^6^ bone marrow-derived MSCs (DM + MSCs mice). During the study period (16 weeks), 45% (11/25) of DM mice and only 8% (2/25) of DM + MSCs mice died or had to be sacrificed because of severe weight loss and cachexia. These animals were excluded from data analysis. DM and DM + MSCs mice remain hyperglycemic, polyuric, hypoinsulinemic, and dyslipidemic compared with normal mice (Table 1).

### 3.1. MSC Administration Prevents Renal Failure

We asked whether MSCs were able to prevent renal failure. In order to address this question renal function was assessed in all experimental groups eight weeks after diabetes induction (before MSC administration) and two and eight weeks after MSC administration. As shown in Table 2, DM mice presented increased albumin excretion rate and plasmatic BUN levels at every time point. After 16 weeks from diabetes induction, they reached values of albuminuria 10 times higher and values of plasmatic BUN two times higher than that observed in normal mice. By contrast, DM + MSCs mice maintained low levels of albuminuria and plasmatic BUN (similar to the pre-MSC administration stage) until the end of the study period.

Plasmatic creatinine levels can be used to show signs of renal function in the end-stage of renal disease [14]. During early-stage post-diabetic induction, the plasmatic level of creatinine did not exhibited significant difference in any experimental group, while this level was significantly higher in DM mice compared with normal mice at the end of the study period (16 weeks after diabetes induction). MSC administration to DM mice maintained the levels of plasmatic creatinine similar to normal mice (Table 2).

### 3.2. MSC Administration Prevents Renal Histopathological Changes

To further characterize the therapeutic effect of MSC administration, we analyzed renal morphology. Renal mass index, determined by the kidney weight to body weight ratio, was significantly higher in DM mice compared with normal mice. A significant decrease of this ratio was observed in DM + MSCs mice eight weeks after MSC administration (Table 1).

**Figure 1:**
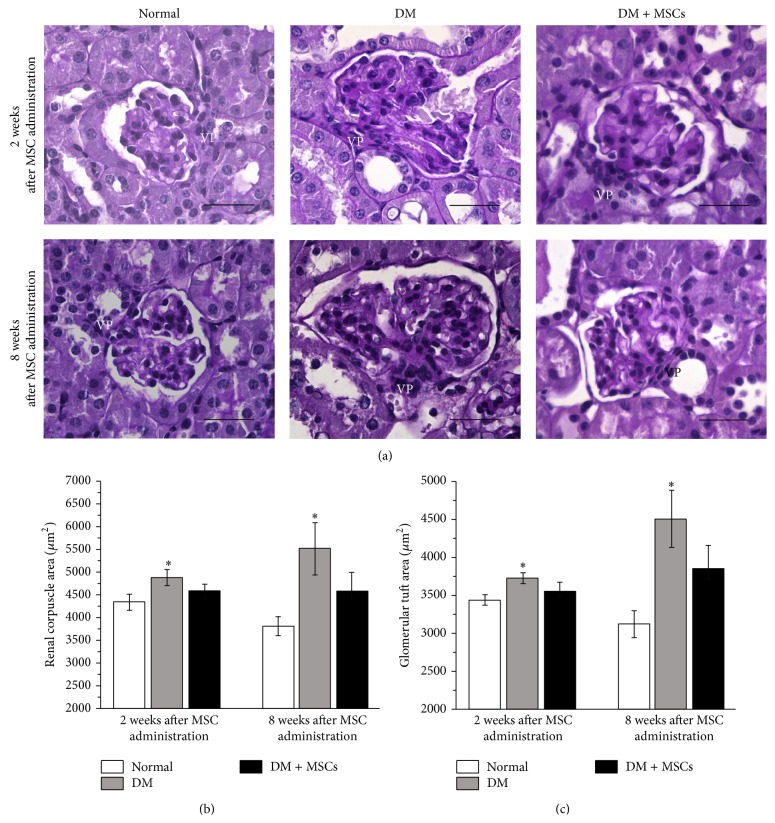
MSC administration reduces glomerular hypertrophic alterations. Representative photomicrographs of kidney sections stained with PAS emphasizing in the glomeruli area, two and eight weeks after MSC administration (a). Quantitative analysis of renal corpuscle area (b) and glomerular tuft area (c) two and eight weeks after MSC administration. Data are presented as mean ± SEM, *n* = 8. ^*∗*^ *p* < 0.05 versus Normal for the same time point. Bar = 25 *μ*m.

Histological alterations in kidney tissue were evaluated by conventional PAS and Masson's trichrome staining and by the presence of the renal damage marker *α*-SMA by immunohistofluorescence. PAS stained sections were analyzed two and eight weeks after MSC administration; kidneys from DM mice showed increased extracellular matrix deposition and glomerular hypertrophy (an increase in renal corpuscle area and glomerular tuft area) compared with kidneys from normal mice (Figures 1(a), 1(b), and 1(c)). Kidneys from DM mice also showed cytoplasmic vacuolation in cortical tubular cells (data not shown). By contrast, kidneys from DM + MSCs mice showed decreased mesangial matrix deposition accompanied with a reduction of the renal corpuscle area and glomerular tuft area, maintaining normal values (Figures 1(a), 1(b), and 1(c)). However, tubular cytoplasmic vacuolations were still observed in the kidneys of DM + MSCs mice (data not shown).

**Figure 2:**
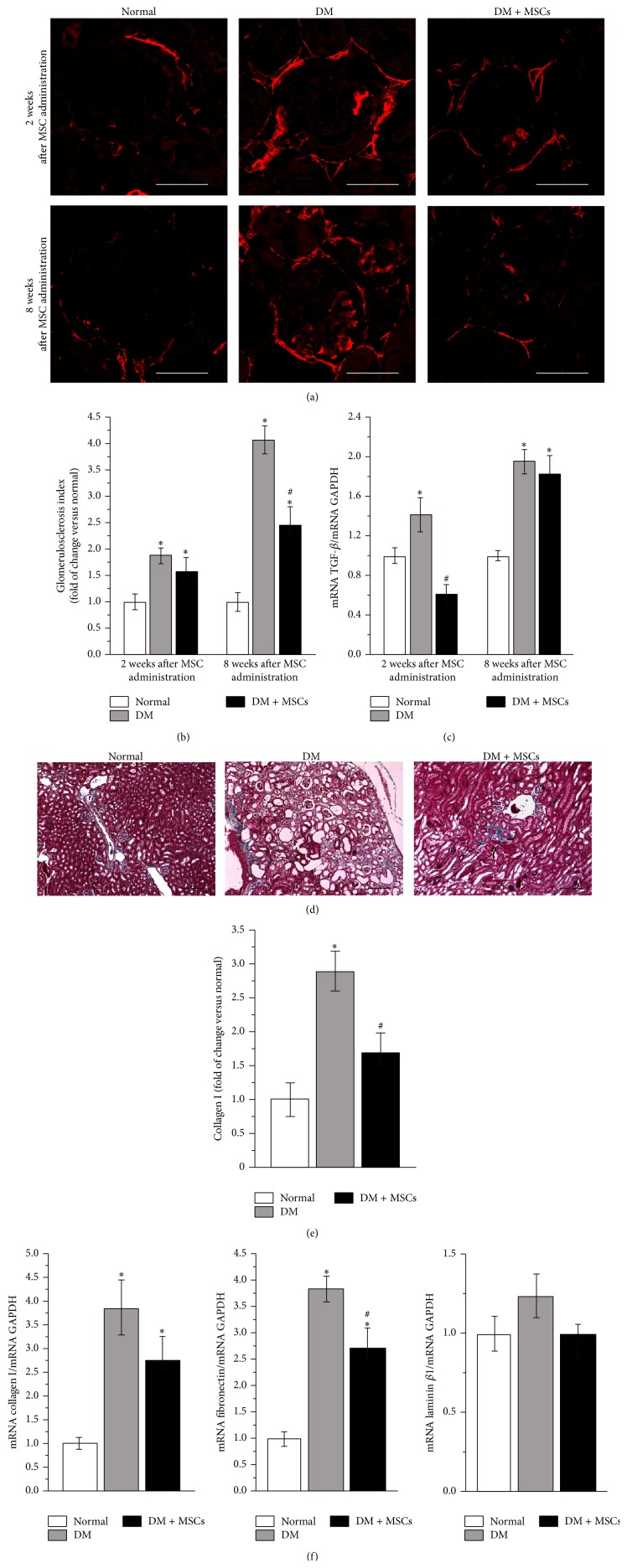
MSC administration prevents renal fibrosis. Representative photomicrographs of kidney sections showing *α*-SMA immunoreactivity in glomeruli, two and eight weeks after MSC administration (a). Quantitative analysis of glomerulosclerosis index evaluated by the percentage of glomeruli exhibiting prominent signal for *α*-SMA in the mesangial area (b). TGF-*β* mRNA levels in renal tissue determined by qRT-PCR (c). Representative photomicrographs of kidney sections stained with Masson's trichrome showing deposition of collagen eight weeks after MSC administration (d). Quantitative analysis of collagen I deposition, evaluated by point-counting method (e). mRNA level of collagen type I, fibronectin, and laminin *β*1 quantified by qRT-PCR eight weeks after MSC administration (f). Data are presented as mean ± SEM, *n* = 8. ^*∗*^ *p* < 0.05 versus Normal; ^#^ *p* < 0.05 versus DM for the same time point. Bar = 150 *μ*m.

The renal damage marker *α*-SMA has been used as an early marker of glomerulosclerosis [33, 40–42]. In accordance with previous reports, immunohistofluorescence signal for *α*-SMA was not detected in normal glomeruli except for smooth muscle cells of efferent and afferent arterioles [14, 33]. Compared with normal mice, the percentage of glomeruli with prominent signal for *α*-SMA in the mesangial area was gradually increased in the kidney of DM mice, leading to an increase in the glomerulosclerosis index (Figures 2(a) and 2(b)). However, the percentage of glomeruli with *α*-SMA immunoreactivity in the kidneys of DM + MSCs mice was significantly reduced at every time point, leading to a reduction in the glomerulosclerosis index (Figures 2(a) and 2(b)). Moreover, the mRNA level of TGF-*β*, a prosclerotic cytokine, was significantly reduced two weeks after MSC administration compared with DM mice (Figure 2(c)).

Eight weeks after diabetes induction Masson's trichromic stained sections from DM mice showed morphological alterations which are signal of chronic damage, such as abundant collagen fiber deposition, loss of brush border, flattening of the epithelia, presence of tubular detritus, and a large quantity of dilated tubules compared with normal mice (Figure 2(d)). On the other hand, kidney sections from DM + MSCs mice showed more conserved morphologies and a significant reduction in the fibrotic area, compared with DM mice (Figures 2(d) and 2(e)). This reduction was also reflected in the mRNA levels of some profibrotic molecules. qRT-PCR studies showed that the expression levels of collagen type I and fibronectin in renal tissue from DM mice were remarkably increased compared with normal mice (Figure 2(f)). Laminin *β*1 expression was also increased in the kidneys from DM mice; however this difference did not reach a statistical significance (Figure 2(f)). All these profibrotic markers showed a tendency to decrease in the kidney of DM + MSCs mice compared with DM mice (Figure 2(f)), suggesting that renal fibrosis was reduced after MSC administration.

### 3.3. MSC Administration Prevents Renal Ultrastructural Changes

**Figure 3:**
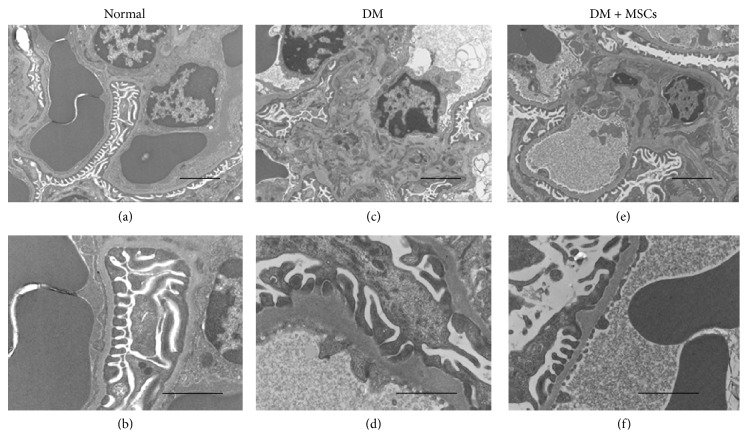
MSC administration prevents renal ultrastructural alterations. Electron photomicrographs of representative glomeruli from normal mice (a and b), DM mice (c and d), and DM + MSCs mice (e and f), showing marked (c) and moderate (f) increase of extracellular mesangial matrix, overt irregularities and thickening of the GBM (d), and persistent “hump like” protrusions (f). Picture magnification: (a), (c), and (e) ×6,000; (b), (d), and (f) ×16,500. Bars magnifications: (a), (c), and (e) 2,000 nm; (b), (d), and (f) 1,000 nm.

At the ultrastructural level, normal kidneys showed scanty amount of extracellular matrix around mesangial cells and glomerular basement membrane (GBM) with a thickness between 150 and 180 nm (Figures 3(a) and 3(b)). Furthermore, the kidneys from DM mice showed marked widening of the GBM (range between 230 and 625 nm) with segmental “hump-like” localized protrusions of the capillary loops, along with up to 35% foot process effacement of the podocytes and a significantly increased in mesangial matrix deposition (Figures 3(c) and 3(d)). By contrast, kidneys from DM + MSCs mice showed less extracellular mesangial expansion than DM mice and normal thickness of the GBM and persistent protrusions of the subepithelial side of the GBM (Figures 3(e) and 3(f)). Thus, MSC administration preserves normal renal ultrastructure.

### 3.4. MSC Administration Maintains the Integrity of the Glomerular Filtration Barrier

The damage caused to the filtration barrier triggers a slow and inexorable decline in renal function that can ultimately result in renal failure. To identify the molecular basis of the renoprotection observed after MSC administration, we evaluated the glomerular capillary lumen, we performed immunostaining for WT-1 and podoplanin, and we measured the expression of nephrin, which are all marker indicatives of the status of the glomerular filtration barrier.

**Figure 4:**
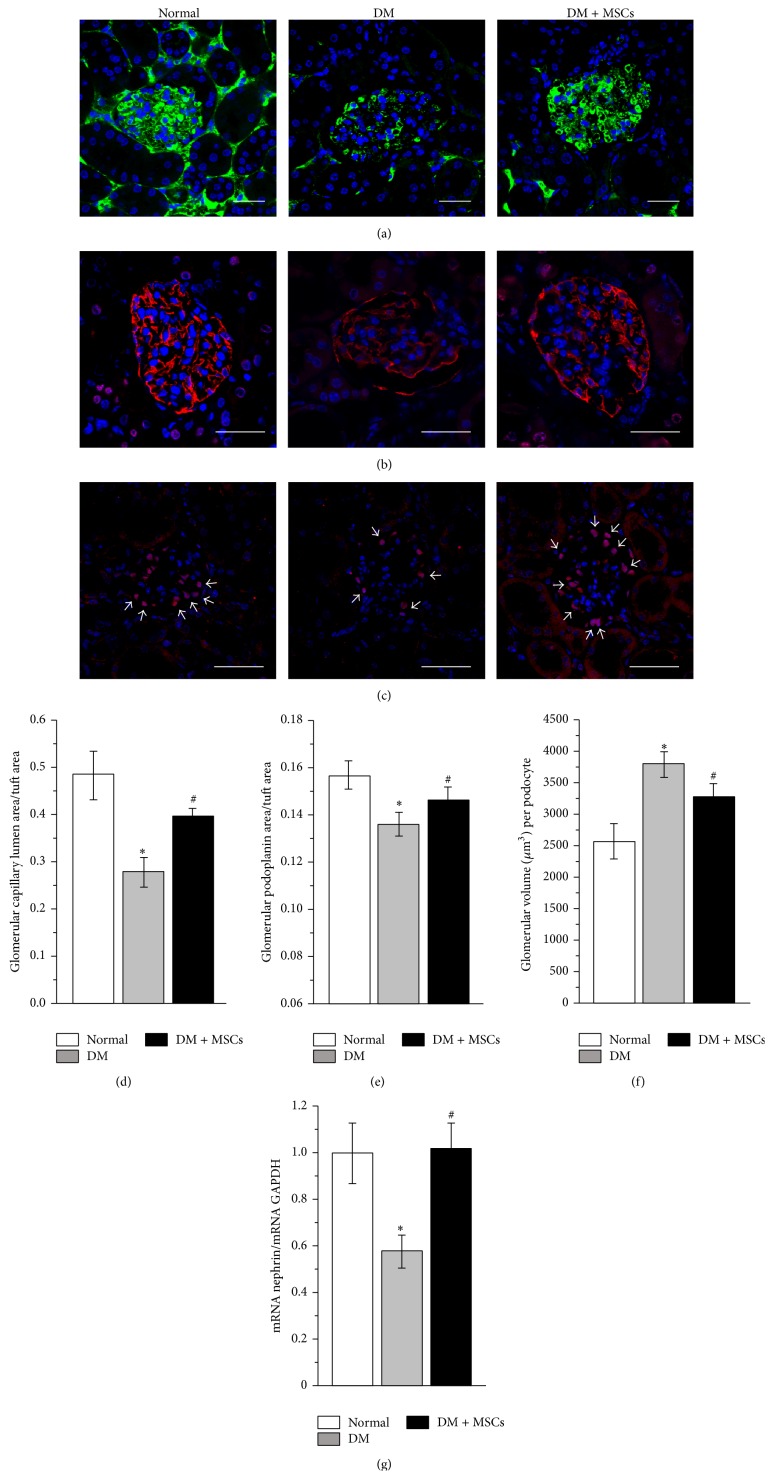
MSC administration maintains the integrity of the glomerular filtration barrier. Representative photomicrographs of kidney sections eight weeks after MSC administration showing glomerular capillary lumen area (green), labeled by the endovenous administration of 2 × 10^6^ MW FITC-dextran (a), glomerular immunoreactivity of podoplanin (red) to evaluate podocyte foot processes integrity (b), and glomerular immunoreactivity of WT-1 (nuclei in red) to evaluate glomerular podocytes (white arrows) (c). In all cases total nuclei were counterstained with DAPI (blue). Quantitative analysis of capillary lumen area normalized by glomerular tuft area (d); glomerular podoplanin area normalized by glomerular tuft area (e); and glomerular volume per podocyte (f). mRNA level of nephrin quantified by qRT-PCR (g). Data are presented as mean ± SEM, *n* = 8. ^*∗*^ *p* < 0.05 versus Normal; ^#^ *p* < 0.05 versus DM for the same time point. Bar = 25 *μ*m.

The ratio of glomerular capillary lumen area/glomerular tuft area was calculated at the end of the study period. In normal mice, the glomeruli capillary network was strongly labeled whereas capillary lumen area was greatly reduced in the glomeruli of DM mice. By contrast, the glomeruli of DM + MSCs mice maintained a strong capillary network similar to normal mice (Figures 4(a) and 4(d)).

Podoplanin is localized on cell membranes of podocytes and is involved in the maintenance of the highly specialized structure of the podocytes and its foot processes, which is essential to the normal functioning of the glomerular filtration barrier [34]. Since quantitative western blotting of glomerular protein extract would not show subtle segmental changes in the expression of podoplanin, we performed immunohistofluorescence to detect the glomerular expression of this marker. At the end of the follow up, there was a focal and segmental loss of podoplanin protein in the glomeruli of DM mice, while MSC administration significantly attenuated this loss (Figures 4(b) and 4(e)). Additionally, the number of podocytes per glomeruli (evaluated by WT-1 staining) was reduced in DM mice; however this difference did not reach statistical significance (data not shown); however, more important than the podocyte number* per se* is the podocyte number in relation to the territory that each podocyte has to cover. Accordingly, the glomerular volume per podocyte was significantly greater in the glomeruli of DM mice compared with normal animals, while in the glomeruli of DM + MSCs mice the glomerular volume per podocyte was significantly reduced compared with DM mice (Figures 4(c) and 4(f)).

Finally, we evaluated the expression of nephrin, which is the principal protein involved in the formation of slit diaphragm in the filtration barrier [43, 44]. In line with the above results, qRT-PCR analysis showed that MSC administration significantly attenuated the diabetic-induced decrease of nephrin expression (Figure 4(g)).

### 3.5. Administered MSCs Do Not Massively Integrate into the Kidneys

**Figure 5:**
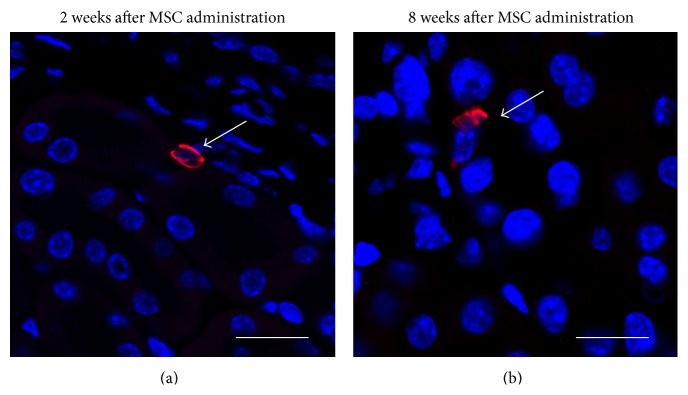
Administered MSCs do not massively integrate into the kidney structure. Two and eight weeks after MSC administration MSC^GFP^ were localized by confocal microscopy. Representative photomicrographs of kidney sections form DM mice showing the localization of MSCs^GFP^, white arrows indicate donor cells immunolabeled with anti-GFP-Alexa555 (red); cell nuclei were counterstain with DAPI (blue). Bars = 15 *μ*m.

In order to evaluate if the therapeutic effect observed after MSC administration could be related to the integration of MSCs into the damaged kidney structures, the renal homing of MSCs^GFP^ was assessed in DN and normal mice, two weeks and eight weeks after MSC administration. Immunohistofluorescence for GFP demonstrated that, in DM mice at both time points, only few MSCs^GFP^ were present in the kidney, mainly around renal tubulointerstitium (Figures 5(a) and 5(b)). No MSCs^GFP^ were found in the kidneys of normal mice (data not shown).

### 3.6. MSC Administration Triggers a Proregenerative Microenvironment

**Figure 6:**
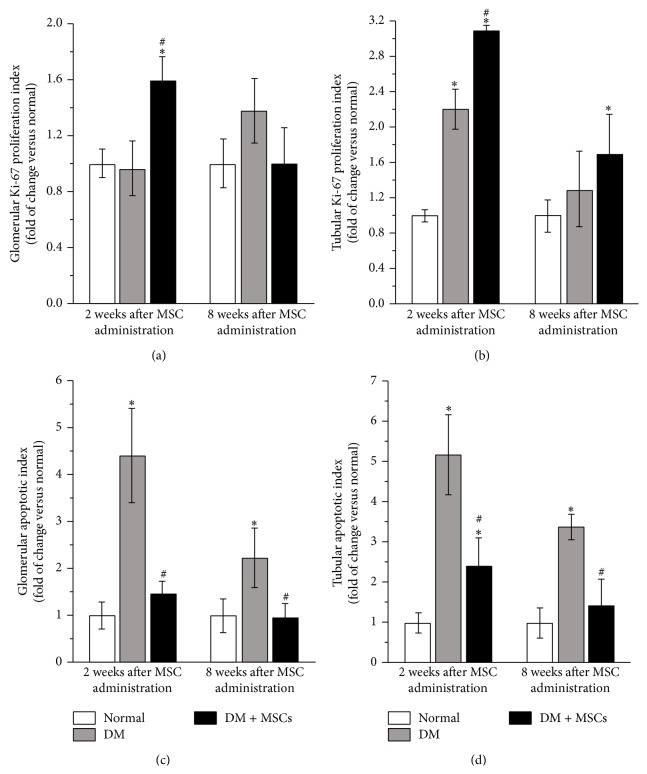
MSC administration induces kidney tissue regeneration. Quantitative analysis of the Ki-67 proliferation index in glomeruli (a) and tubules (b) two and eight weeks after MSC administration. Quantitative analysis of the apoptotic index in glomeruli (c) and tubules (d) two and eight weeks after MSC administration. Data are presented as mean ± SEM, *n* = 8, ^*∗*^ *p* < 0.05 versus Normal; ^#^ *p* < 0.05 versus DM for the same time point.

To understand if the therapeutic effects observed after MSC administration could be related to the generation of a proregenerative microenvironment, the rate of cell proliferation in the kidneys was determined by the quantification of the nuclear expression of Ki-67 and glomerular and tubular sections were analyzed independently. The glomeruli of DM + MSCs mice presented higher proliferation rate two weeks after MSC administration compared with normal and DM mice (Figure 6(a) and Supplementary Figure 1). At tubular level, DM mice showed a significant increase in the proliferation rate compared with normal mice, probably associated with a compensatory response to chronic injury. Nevertheless, the administration of MSCs significantly increased this response (Figure 6(b) and Supplementary Figure 1).

The apoptotic index was evaluated in glomerular and tubular sections. As expected, DM mice showed a marked increase in the number of apoptotic nuclei in both areas (Figures 6(c) and 6(d) and Supplementary Figure 2). Nevertheless, this increase was significantly reduced in the kidney of DM + MSCs mice after two weeks and eight weeks after MSC administration.

**Figure 7:**
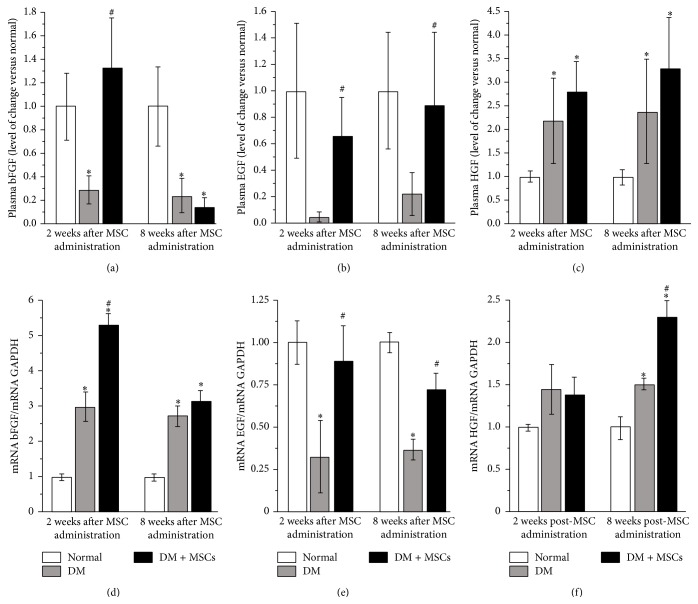
MSC administration increases local and systemic levels of proregenerative factors. Quantitative analysis of plasmatic levels of bFGF (a), EGF (b), and HGF (c) two and eight weeks after MSC administration determined by Luminex Multiplex system. Renal mRNA levels of bFGF (d), EGF (e), and HGF (f) quantified by qRT-PCR, two and eight weeks after MSC administration. Data are presented as mean ± SEM, *n* = 8. ^*∗*^ *p* < 0.05 versus Normal; ^#^ *p* < 0.05 versus DM for the same time point.

MSCs are known to produce, both* in vitro* and* in vivo*, a broad range of trophic factors that have been associated with tissue regeneration by induction of proliferation and reduction of apoptosis [45]. Systemic bFGF and EGF levels were markedly reduced in DM mice compared with normal mice, while the administration of MSCs restored the normal plasmatic level of both regenerative factors (bFGF two weeks after MSC administration and EGF two and eight weeks after MSC administration) (Figures 7(a) and 7(b)). Plasmatic HGF levels were increased both in DM mice and DM + MSCs mice compared with normal mice (Figure 7(c)) and plasmatic levels of VEGF and ET1 were unchanged in any experimental condition (data not shown).

The renal tissue has high regeneration capacity associated, at least in part, to the secretion of trophic factors [46]. To evaluate if MSC administration could induce a proregenerative microenvironment, the local expression of trophic factors was evaluated by qRT-PCR. In accordance with plasmatic levels, the renal expression of EGF decreased significantly in diabetic animals and MSC administration restored normal EGF mRNA levels (Figure 7(e)). Additionally, diabetic mice presented high mRNA levels of bFGF and HGF in the kidney; however, MSC administration significantly enhanced this response (Figures 7(d) and 7(f)).

### 3.7. MSC Administration Reduces Oxidative Stress Damage

**Figure 8:**
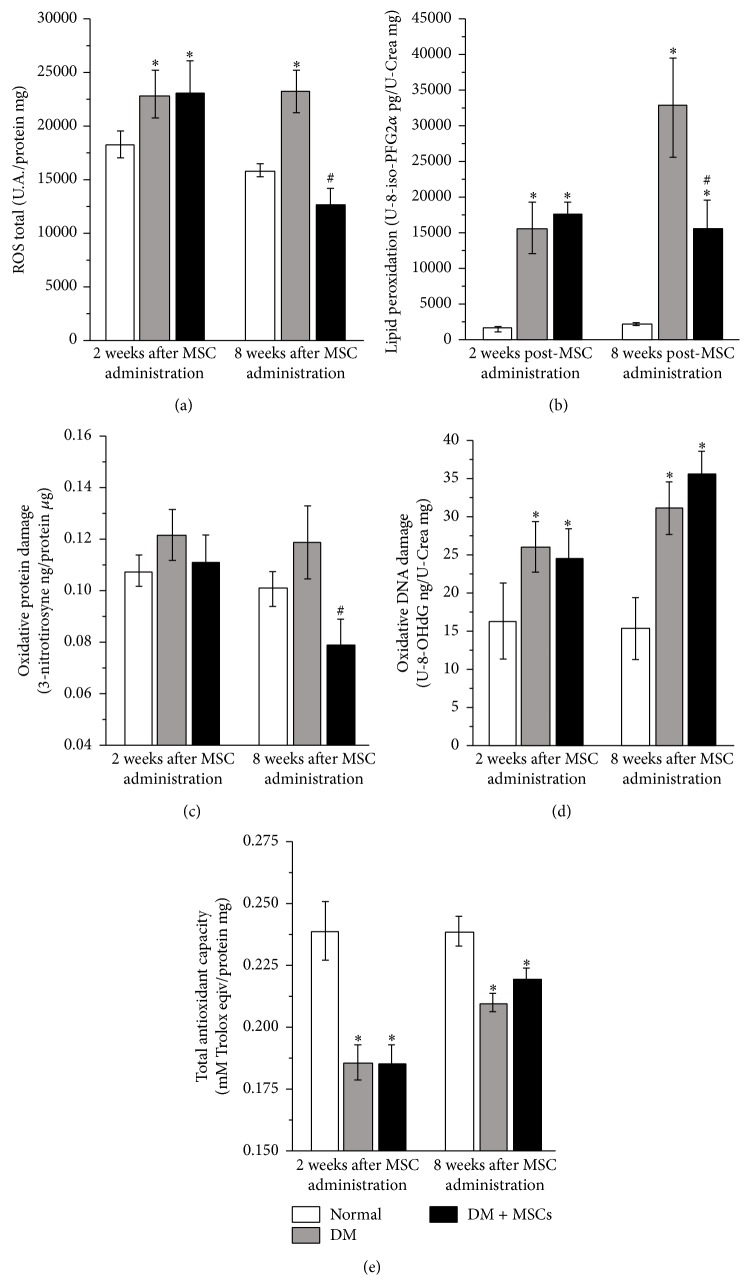
MSC administration reduces oxidative stress. Quantitative analysis of total reactive oxygen species expressed as total ROS per mg of kidney proteins (a); lipid peroxidation level expressed as pg of urinary 8-iso-PFG2*α* per mg of urinary creatinine (b); oxidative protein damage level expressed as ng of 3-nitrotyrosine per *μ*g of kidney proteins (c); oxidative DNA damage level expressed as ng of urinary 8-OHdG per mg of urinary creatinine (d); and total antioxidant capacity expressed as mM of Trolox equivalents per mg of kidney proteins (e) two and eight weeks after MSC administration. Data are presented as mean ± SEM, *n* = 8. ^*∗*^ *p* < 0.05 versus Normal; ^#^ *p* < 0.05 versus DM for the same time point.

Oxidative stress plays an important role at both early and late stages of DN [6] and it has been postulated that MSCs could efficiently scavenge reactive species [47]. Total oxidative species were evaluated in renal tissue, two and eight weeks after MSC administration. The diabetic condition induced a significant increase in the amount of total oxidative species in the kidney that was significantly reduced eight weeks after MSC administration (Figure 8(a)). The reduction in ROS level was correlated with a significant reduction in lipid peroxidation level (Figure 8(b)) and protein oxidative damage (Figure 8(c)). By contrast, the DNA oxidative damage remained significantly elevated irrespective of MSC administration (Figure 8(d)).

Total antioxidant capacity was reduced in DM mice, with a trend to be higher in the treated mice eight weeks after MSC administration (Figure 8(e)).

### 3.8. MSC Administration Prevents Renal Inflammation

**Figure 9:**
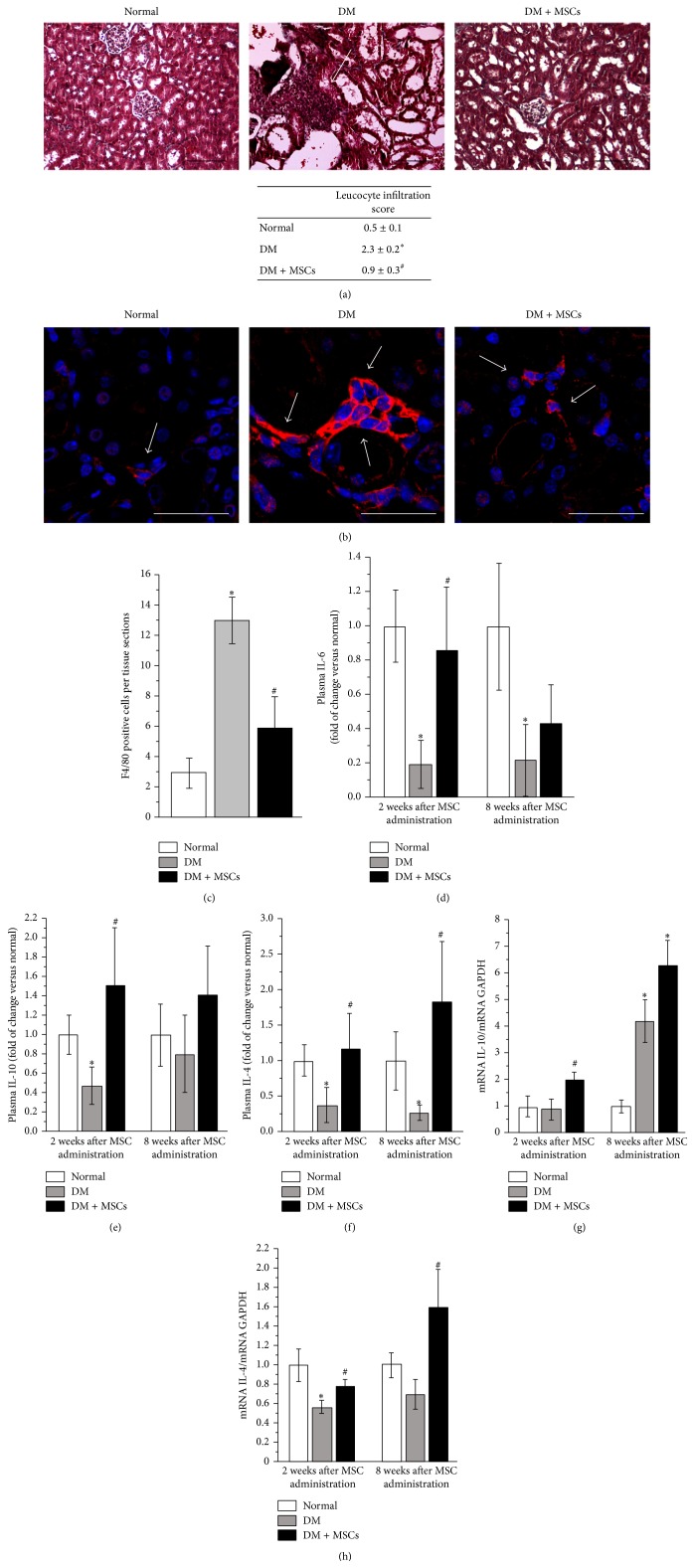
MSC administration prevents renal inflammation. Representative photomicrographs of kidney sections showing inflammatory infiltrates (white arrow) eight weeks after MSC administration. Bar = 100 *μ*m (a). Representative photomicrographs of kidney sections showing macrophages (white arrows) (F4/80-red) eight weeks after MSC administration. Barr = 40 *μ*m (b). Nuclei were counterstained with DAPI (blue). Quantitative analysis of macrophages per tissue section eight weeks after MSC administration (c). Quantitative analysis of plasmatic levels of the anti-inflammatory cytokines IL-6 (d), IL-10 (e), and IL-4 (f) determined by the Luminex Multiplex system two and eight weeks after MSC administration. Renal mRNA levels of IL-10 (g) and IL-4 (h) quantified by qRT-PCR, two and eight weeks after MSC administration. Data are presented as mean ± SEM, *n* = 8, ^*∗*^ *p* < 0.05 versus Normal; ^#^ *p* < 0.05 versus DM for the same time point.

The inflammatory influx is present in the diabetic kidney and is one of the driving forces to induce tissue damage [48, 49]. Multifocal inflammatory cellular foci were observed in the kidney of DM mice (Figure 9(a)). By contrast, no leukocyte infiltration foci were observed in the kidney of DM + MSCs mice (Figure 9(a)). Since macrophages account for most of the infiltrating leukocytes (>90%) [30] and they correlate with increased levels of albuminuria and plasma creatinine [50, 51], F4/80 cells in renal tissue were evaluated. In accordance with the above result, diabetic animals presented an increased number of macrophages per sections, and this was significantly reduced by MSC administration (Figures 9(b) and 9(c)).

MSCs have been recognized as immunomodulatory cells with potent immunosuppressive properties [13]. Based on these properties, we measured the serum levels and the renal expression of cytokines related to proinflammatory and anti-inflammatory responses.

The administration of MSCs did not change the plasmatic level of the proinflammatory cytokines TNF-*α* and IL-1*β* (data not shown). However, MSC-treated mice showed a plasmatic cytokine profile compatible with a Th2 polarized immune response, since plasmatic levels of IL6, IL-4, and IL-10 were restored, compared with DM mice (Figures 9(d), 9(e), and 9(f)).

The same pattern was observed in renal tissues, since MSC-treated animals showed a renal cytokine expression profile compatible with a Th2 polarized immune response, with a high expression of IL-4 and IL-10 (Figures 9(g) and 9(h)). Therefore, MSC administration induces a protective microenvironment.

## 4. Discussion

The major findings of this study were that MSC administration preserved renal function and ameliorate histopathological alterations in an animal model of severe diabetes mellitus. The possible mechanisms underlying these effects involved the temporal inhibition of the TGF-*β* expression and the triggered of a proregenerative microenvironment, which include the production of protective trophic factors and the reduction of both the oxidative stress damage and the proinflammatory response in the kidney.

Using an animal model of nonimmunological severe diabetes mellitus induced by the administration of a single high dose of STZ (200 mg/kg), we previously showed that the administration of multiple doses of MSCs was able to prevent albuminuria development despite the persistence of hyperglycaemia [21] suggesting a direct effect of MSCs in the prevention of renal damage. In the present study, using the same animal model we further characterize the effects of MSC administration on renal function and structure, along with the possible mechanisms associated with the therapeutic effects.

MSC administration effectively reduced urinary albumin excretion and plasmatic BUN and creatinine levels. The preservation of renal function lasted at least up to the end of the follow-up study (eight weeks after MSC administration). This improvement in renal function was clearly associated with the preservation of renal structure, since untreated diabetic mice presented glomerular hypertrophy, mesangial expansion, tubular dilatation, glomerulosclerosis, tubulointerstitial fibrosis, and GBM thickening, while the administration of MSCs prevented all these histopathological alterations.

TGF-*β* upregulation is considered one of the main factors involved in the development and progression of DN [8]. It stimulates renal cell hypertrophy and extracellular matrix deposition, which causes GBM thickening, glomerular capillary occlusion, and may promote podocyte apoptosis or detachment [35]. In the present severe diabetes mellitus model, we observed an early reduction in the renal expression of TGF-*β*1 after MSC administration, suggesting that glomerulosclerosis and renal interstitial fibrosis were efficiently prevented. The same observation was made in diabetic spontaneous hypertensive rats where the inhibition of TGF-*β* expression by Tranilast, an antiallergic drug that impedes the release of cytokines from immune cells, led to a reduction in glomerular hyperfiltration and glomerular tuff area expansion despite persistent hyperglycaemia [52]. In the same line,* in vitro* and* in vivo* studies showed that MSCs could inhibit the upregulation of TGF-*β* expression stimulated by high glucose in mesangial cells by the secretion of trophic factors [53]. Thus, we can assume that the early modulation of TGF-*β* expression after MSC administration is related, at least in part, to a reduction in kidney fibrosis.

Under diabetic condition, high glucose levels compromise the integrity of the glomerular filtration barrier via effacement of the podocyte foot processes and loss of podocytes from the glomerular basement membrane. The damage caused to the filtration barrier triggers a slow and inexorable decline in renal function that can ultimately result in renal failure [54–56]. Podoplanin is a glycoprotein expressed on the base of podocyte foot processes and is involved in the maintenance of the highly specialized structure of the podocytes and its foot processes during hypertrophy [34]. In great contrast to the general pattern of most of the podocyte-associated proteins, podoplanin has showed changes in expression with clear temporal association to the development of proteinuria [57]. In the same way, nephrin is the principal protein involved in the formation of slit diaphragm of the filtration barrier [43, 44, 58, 59]. To identify the molecular basis of the renoprotection observed after MSC administration, we performed immunostaining for WT-1 and podoplanin and evaluated the renal expression of nephrin, which are indicatives of the status of the glomerular filtration barrier. At the end of the study period, untreated mice showed a significant increase in the glomerular volume per podocyte that was accompanied by focal and segmental loss of glomerular podoplanin protein expression, while MSC administration restored the normal level of podoplanin, nephrin, and the normal glomerular volume per podocyte.

It has been hypothesized that the expansion of the glomerular tuft requires the adaptation of the glomerular epithelial cells to cover a wide area of glomerular capillary wall, a process that requires the reorganization of the actin cytoskeleton and a higher podocyte workload. The increased workload may lead to podocyte damage and eventually podocyte loss [60]. Several studies have reinforced the point that the primary issue of concern is probably not the podocyte number per se but the podocyte number in relation to the territory that each podocyte has to cover designated as glomerular volume per podocyte [61–64]. The glomerular hypertrophy observed in DM mice added to the diabetic microenvironment could result in an increased podocyte workload, followed by the loss of the filtration barrier integrity and the development of albuminuria. By contrast, the preservation of the integrity of the glomerular filtration barrier in the kidney of MSC-treated mice could be associated to the reduction of glomerular hypertrophy and the production of trophic factors. According to this,* in vitro* studies showed that human adipose-derived MSCs could prevent podocyte injury induced by high glucose mainly through secreting EGF [65]. Additionally, MSCs contribute to podocyte regeneration by trophic factor secretion when administered in an animal model of Alport syndrome [66].

In this study we found that after MSC administration, a small amount of donor cells were present in the kidney, mainly in the tubulointerstitial space. The selectivity of this cell recruitment was supported by the fact that no donor cells were detected when MSCs^GFP^ were administered into normal mice. Thus, in agreement with already published data, MSC recruitment to kidney depends on the presence of injury [67]. Therapeutics effects with reduced MSCs homing have been reported in other studies of tissue damage [24, 68–71]. So it is likely reasonable to speculate that MSCs protected the diabetic kidney primarily via paracrine mechanisms.

In our study, the improvement of functional parameters was associated with an acceleration of the regeneration phase, as demonstrated by higher amounts of positive Ki-67 nuclei, and increased expression of trophic factors in the kidney of MSC-treated mice. Additionally, this recovery was correlated with an important inhibition of the apoptotic process.

It is well recognized that MSCs can secrete a broad range of trophic factors, such as HGF, bFGF, IGF-1, and EGF, all known to improve the renal function in animal models of kidney injury, due to their antiapoptotic, mitogenic, and morphogenic activities on intrarenal cells [45, 66]. Based on these observations, we speculate that the mechanisms that mediate protective effects after MSC administration might be primarily related to the paracrine secretion of these factors, creating a proregenerative microenvironment that could induce the endogenous regeneration of the tissue.

In our severe diabetic animal model we showed a systemic increase in the plasmatic levels of bFGF (two weeks after MSC administration) and EGF and HGF (2 and 8 weeks after MSC administration) and also a local increase in the expression levels of these proregenerative factors. Both* in vitro* and* in vivo* experiments, EGF can reduce apoptosis and induce the repair of the damage renal tubular epithelial cells [72]. According to this, it has been found in an* in vitro* study that human adipose-derived MSCs could prevent podocytic apoptosis induced by high glucose mainly through secreting EGF [65] and that MSCs contribute to podocyte regeneration by trophic factor secretion when administered in an animal model of Alport syndrome [66]. Moreover, bFGF can also inhibit the apoptotic process and accelerate renal tissue regeneration when is exogenously administrated [73]. The effect of bFGF on renal injury is particularly important since it has been suggested that bFGF can induce the expression of several tubulogenic/epitheliogenic and angiogenic proteins like Pax-2, bone morphogen protein-7, Noggin, Smad 1-5-8, p-Smad, and hypoxia-inducible factor-1, which in turn are responsible for kidney regeneration [73]. On the other hand, renal damage is exacerbated when bFGF is inhibited [74].

Glucose is the primary fuel source for ROS generation [6] and it is known that oxidative stress condition increase the vulnerability of renal cells [6]. We showed that after MSC transplantation renal cell death and lipid peroxidation were significantly reduced, despite the sustained high blood glucose levels.

There is growing evidence indicating that MSCs can efficiently control oxidative stress. Previously we have demonstrated that the low susceptibility of MSCs to the harmful effect of ROS correlates with the ability of these cells to effectively scavenge peroxide and peroxynitrite due to constitutive expression of the enzymes superoxide dismutases 1 and 2, catalase, and glutathione peroxidase [47]. Moreover, MSCs possess the main enzymatic mechanisms to detoxify reactive species, guaranteeing an efficient regulation of oxidative stress [75].

Previous reports of clinical and preclinical studies have indicated that inflammatory cytokines play an important role in the onset and progression of DN in which macrophages infiltrate the kidney and produce a proinflammatory microenvironment [5]. In this sense, it has been demonstrated that anti-inflammatory treatments and immunotherapy could attenuate podocyte injury, renal fibrosis, and alleviate albuminuria [14, 76]. Here we showed that the number of infiltrating macrophages in diabetic mice was markedly reduced after MSC administration [13]. Additionally, at molecular level a significant reduction in the plasmatic levels of IL-4, IL-10, and IL-6 was observed in untreated diabetic mice compared with normal animals. These levels were normalized in MSC-treated mice. IL-4 and IL-10 are the main anti-inflammatory and immunosuppressive cytokines which are produced by several types of immune cells such as regulatory T cells and Th2 lymphocytes [5]. These cytokines play a key role in the regulation of immune responses acting as potent deactivator of the synthesis of monocyte/macrophage proinflammatory cytokine and inhibiting leukocyte infiltration, inflammation, and tissue damage [14]. In different animal models, it has been reported that MSC administration reduces macrophage infiltration into the target tissues [14, 77] and switches macrophages from M1 to M2 phenotype contributing to tissue homeostasis [78]. MSCs can secrete IL-4 and IL-10 generating a protective microenvironment that might avoid the immunological destruction of renal cells [9]. Strikingly, plasmatic IL-6 reached normal levels two weeks after MSC administration. This fast recovery may be due in part to the release of IL-6 by the administered MSCs. IL-6 is a pleiotropic cytokine that regulates immune responses and inflammatory reactions. Djouad et al. showed that murine MSCs secrete high level of IL-6 and this level is directly correlated to the inhibition of the proliferation of T-cells [79].

In the kidney of MSC-treated mice, we also observed a skewing toward a Th2 profile, as demonstrated by the increased expression of IL-4 and IL-10. The local attenuation of inflammation has been associated to a reduction in fibrosis and the generation of a protective microenvironment that supports the tissue regeneration [13, 14, 77, 80].

## 5. Conclusions

In summary, our study provides further evidence that MSCs can not only exert antialbuminuric effects but also prevent glomerular capillary occlusion and maintain podocyte density and foot process in the glomerular filtration barrier. In this animal model of severe diabetes mellitus, the improvement of the glomerular function and the morphological recoveries observed after MSC administration could be mediated, at least in part, by the promotion of endogenous regeneration of the kidney tissue due to changes in the local microenvironment and the inhibition of oxidative stress damage and inflammatory reactions skewing a Th2 profile.

We think that the successful treatment of DN with MSCs demonstrated here holds a substantial promise for the development of novel MSC-based interventions that can prevent the progression of DN. These findings should be confirmed in clinical trial, since MSC transplantations in human patients is feasible and safe.

## Supplementary Material

Determination of Renal Mitotic and Apoptotic Indexes. Mitotic index was determined by immunohistofluorescence using an anti-Ki-67 antibody. Positive nucleus indicate cell cycle activity (G1, S, G2, or M phase). Apoptotic cells in kidney tissue slices were visualized using the DeathEnd Fluorometric TUNEL System (Promega), following the manufacture´s protocol. The nuclei were counterstained with DAPI and fluorescence was evaluated by confocal microscopy.
Supplementary Figure 1: MSC administration induces renal cells proliferation. Representative photomicrographs of kidney sections showing Ki-67 immunoreactivity (red) in glomeruli and tubules two and eight weeks post-MSC administration. Nuclei were counterstained with DAPI (blue). White arrows indicate Ki-67 positive nuclei. Barr= 25µm.
Supplementary Figure 2: MSC administration prevent apoptosis in renal cells. Representative photomicrographs of kidney sections showing TUNEL staining (green) in glomeruli and tubules two and eight weeks post-MSC administration. Nuclei were counterstained with DAPI (blue). White arrows indicate TUNEL stained nuclei. Barr= 25 µm.
Supplementary Table 1: RT-PCR specific primers and characteristics of amplicons.

## Figures and Tables

**Table 1:** MSC administration did not improve diabetic condition.

	Before MSC administration		8 weeks after MSC administration		
	Normal	DM	Normal	DM	DM + MSCs
Body weight (g)	24.2 ± 0.4	20.2 ± 0.3^*∗*^	26.9 ± 0.5	21.4 ± 0.5^*∗*^	22.2 ± 0.5^*∗*^
Plasma insulin (*μ*g/L)	0.61 ± 0.12	0.03 ± 0.01^*∗*^	0.51 ± 0.10	0.07 ± 0.03^*∗*^	0.07 ± 0.03^*∗*^
Blood glucose (mg/dL)	171 ± 2	715 ± 49^*∗*^	183 ± 11	766 ± 68^*∗*^	740 ± 40^*∗*^
Serum cholesterol (mg/dL)	97 ± 10	173 ± 20^*∗*^	146 ± 46	291 ± 32^*∗*^	253 ± 52^*∗*^
Serum triglycerides (mg/dL)	72 ± 14	198 ± 35^*∗*^	97 ± 39	337 ± 55^*∗*^	217 ± 39^*∗*^
Kidney weight (mg)	158 ± 4	217 ± 9^*∗*^	177 ± 5	311 ± 15^*∗*^	278 ± 14^*∗*#^
Kidney/body weight (mg/g)	5.7 ± 0.2	8.9 ± 0.3^*∗*^	6.6 ± 0.2	15 ± 0.7^*∗*^	12.6 ± 0.9^*∗*#^
Urine volume (mL/24 h)	1.3 ± 0.1	10.0 ± 1.6^*∗*^	2.4 ± 0.9	16.8 ± 2.3^*∗*^	16.3 ± 2.2^*∗*^

Physical and biochemical parameters of experimental animals. Before MSC administration (eight weeks after diabetes induction), mice received 200 *μ*L of 5% mice plasma (DM) or 0.5 × 10^6^ MSCs resuspended in 200 *μ*L of 5% mice plasma (DM + MSCs) via the tail vein. All biochemical parameters were evaluated in urine and plasma samples obtained after four hours of fasting. Data are presented as mean ± SEM, *n* = 12. ^*∗*^ *p* < 0.05 versus Normal; ^#^ *p* < 0.05 versus DM for the same time point.

**Table 2:** MSC administration prevents renal failure.

	Before MSC administration		2 weeks after MSC administration			8 weeks after MSC administration		
	Normal	T1DM	Normal	T1DM	T1DM + MSC	Normal	T1DM	T1DM + MSC
Urinary albumin excretion (U-Alb *μ*g/U-Crea mg)	6.4 ± 1.4	35.0 ± 3.1^*∗*^	6.7 ± 1.1	40.2 ± 4.3^*∗*^	27.4 ± 4^*∗*#^	7.2 ± 0.8	62.2 ± 9.5^*∗*^	30.9 ± 2.5^*∗*#^
Blood urea nitrogen (mg/dL)	30.1 ± 2	40.2 ± 3.3^*∗*^	32 ± 1.8	42.1 ± 2.2^*∗*^	45.5 ± 4.7^*∗*^	31.9 ± 2.5	67.3 ± 3.8^*∗*^	46.5 ± 3.4^*∗*#^
Plasma cystatin C (mg/L)	0.07 ± 0.01	0.11 ± 0.01^*∗*^	0.06 ± 0.01	0.11 ± 0.02^*∗*^	0.08 ± 0.01	0.06 ± 0.02	0.13 ± 0.02^*∗*^	0.08 ± 0.01^#^
Plasma creatinine (mg/L)	2.26 ± 0.06	2.29 ± 0.08	2.30 ± 0.07	2.27 ± 0.06	2.30 ± 0.13	2.28 ± 0.07	2.60 ± 0.05^*∗*^	2.29 ± 0.05^#^

Markers of renal function were evaluated before MSC administration (eight weeks after diabetes induction) and after two and eight weeks after MSC administration. All animals were housed in metabolic cages to collect urine for albuminuria determinations; blood was collected and used to measure plasmatic creatinine and urea nitrogen levels. Data are presented as mean ± SEM, *n* = 12. ^*∗*^ *p* < 0.05 versus Normal; ^#^ *p* < 0.05 versus DM for the same time point.

## Abbreviations

Alb: Albumin
Crea: Creatinine
bFGF: Basic fibroblast growth factor
BUN: Blood urea nitrogen
DAPI: 4′,6-Diamino-2-fenilindol
DM: Severe diabetes mellitus
DN: Diabetic nephropathy
EGF: Epidermal growth factor
GAPDH: Glyceraldehyde-3-phosphate dehydrogenase
GBM: Glomerular basement membrane
GFP: Green fluorescent protein
HGF: Hepatocyte growth factor
MSCs: Mesenchymal stem cells
Nphs: Nephrin
PAS: Periodic acid Schiff
PBS: Phosphate-buffered saline
ROS: Reactive oxygen species
STZ: Streptozotocin
TGF-*β*: Transforming growth factor *β*
TUNEL: Terminal uridine nucleotide end-labeling
VP: Vascular pole
WT-1: Wilms' tumor protein 1
*α*-SMA: Smooth muscle actin *α*.
