# Supplementary material for: Proregenerative Microenvironment Triggered by Donor Mesenchymal Stem Cells Preserves Renal Function and Structure in Mice with Severe Diabetes Mellitus
Source: Biomed Res Int. 2015 Jun 8;2015:164703. doi: 10.1155/2015/164703 (PMC4475763; doi:10.1155/2015/164703)

Supplementary Table 1. RT-PCR specific primers and characteristics of amplicons.

Gene	Nº GenBank access	Sense (5'→3')	Antisense (5'→3')	Amplicon size (bp)	Amplicon Tm (ºC)
collagen type I	NM_007742	AGAACATCACCTATCACTGCAAGA	GTGGTTTTGTATTCGATGACTGTCT	205	89
TGF-beta1	NM_011577	TGACGTCACTGGAGTTGTACGG	GGTTCATGTCATGGATGGTGC	170	88
fibronectin	NM_010233.1	AGACCATACCTGCCGAATGTAG	GAGAGTTTCTGTCCTGTAGAG	129	86
Laminin-beta 1	NM_008482.2	AGACCCGAAGAAAAGACAGGC	CCATAGGGCTAGGACACCAAA	124	90
bFGF	NM_008006	GCGACCCACACGTCAAACTA	TCCATCTTCCTTCATAGCAAGGT	101	84
EGF	NM_010113	TCTCGGATTGACCCAGAT	CCCAGAACACCTTCCTCTCT	192	84
HGF	NM_010427	AAGCAATCCAGAGGTACGCTAC	TGCCATCAGGATTGCGGCAATA	224	87
IL-4	NM_021283	ACTTGAGAGAGATCATCGGCA	AGCTCCATGAGAACACTAGAGTT	208	86
IL-6	NM_031168	ATCCAGTTGCCTTCTTGGGACTGA	TAAGCCTCCGACTTGTGAAGTGGT	134	86
IL-10	NM_010548	AGGGTTACTTGGGTTGCCAA	CACAGGGGAGAAATCGATGA	174	87
Nphs	NM_01945	CAGCGATGATGCGGAGTACG	CAGCTACCCAGGTAACTGTGC	149	85
GAPDH	XM_001474390	ACTCCACTCACGGCAAATTC	TCTCCATGGTGGTGAAGACA	171	88

Supplemantary Figure 1

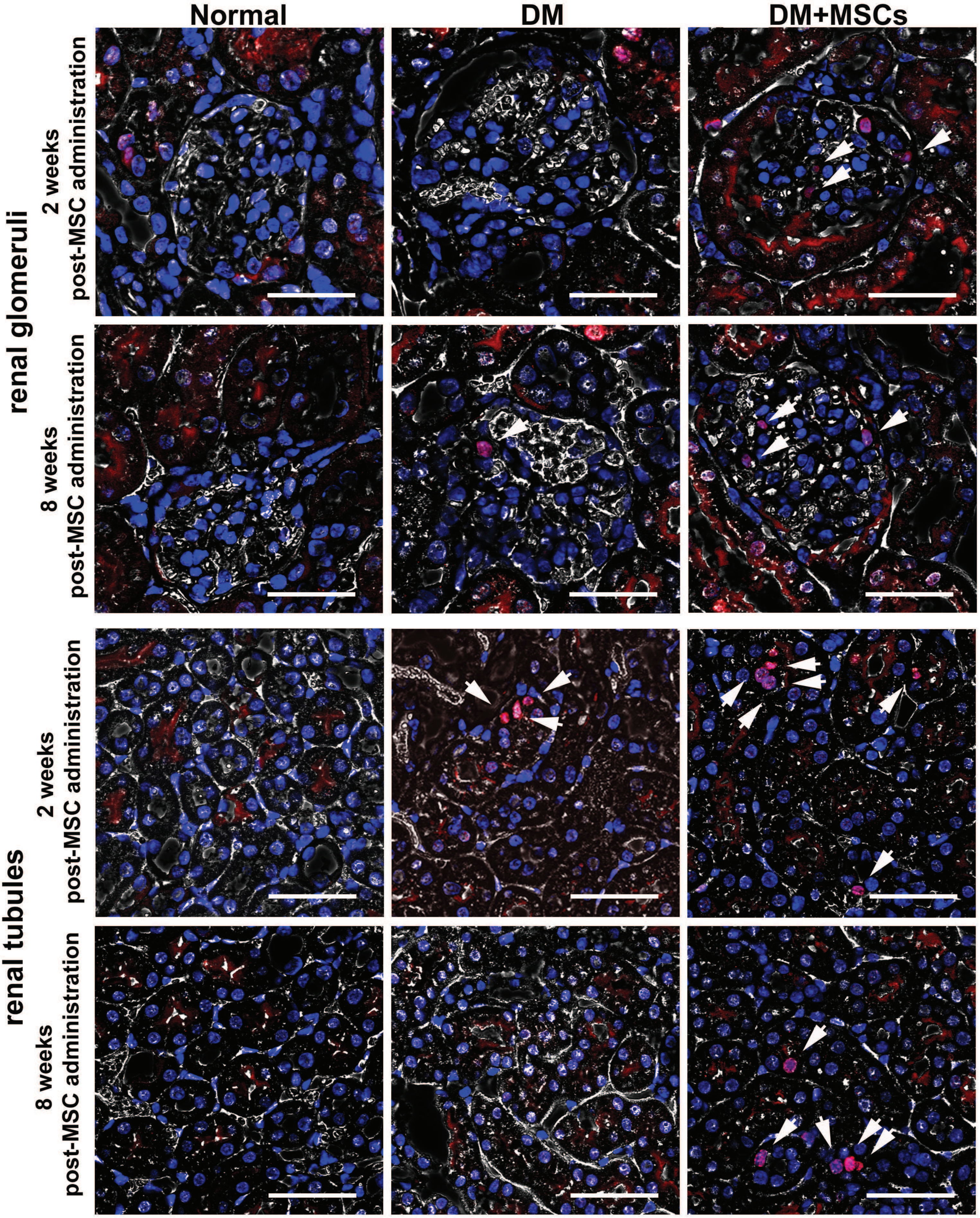

Supplemantary Figure 2

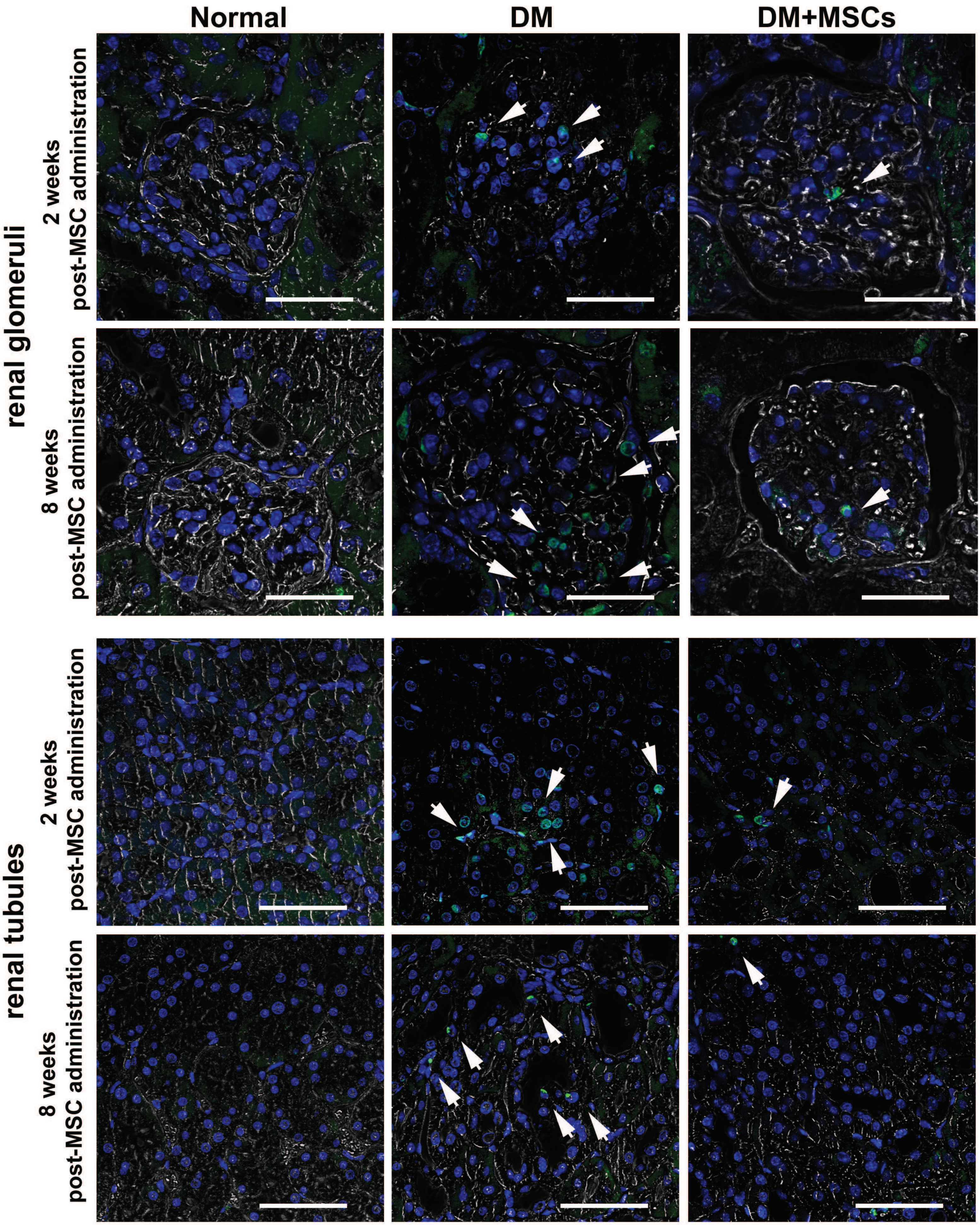

Supplement: Supplementary file 1 — Determination of Renal Mitotic and Apoptotic Indexes. Mitotic index was determined by immunohistofluorescence using an anti-Ki-67 antibody. Positive nucleus indicate cell cycle activity (G1, S, G2, or M phase). Apoptotic cells in kidney tissue slices were visualized using the DeathEnd Fluorometric TUNEL System (Promega), following the manufacture´s protocol. The nuclei were counterstained with DAPI and fluorescence was evaluated by confocal microscopy. Supplementary Figure 1: MSC administration induces renal cells proliferation. Representative photomicrographs of kidney sections showing Ki-67 immunoreactivity (red) in glomeruli and tubules two and eight weeks post-MSC administration. Nuclei were counterstained with DAPI (blue). White arrows indicate Ki-67 positive nuclei. Barr= 25µm. Supplementary Figure 2: MSC administration prevent apoptosis in renal cells. Representative photomicrographs of kidney sections showing TUNEL staining (green) in glomeruli and tubules two and eight weeks post-MSC administration. Nuclei were counterstained with DAPI (blue). White arrows indicate TUNEL stained nuclei. Barr= 25 µm. Supplementary Table 1: RT-PCR specific primers and characteristics of amplicons. [file 164703.f1.pdf]
